# Renal and Bladder Tuberculosis1Read at the semi-annual meeting of the Medical Society of the County of Chautauqua, at Jamestown, December 17, 1901.

**Published:** 1902-02

**Authors:** J. Henry Dowd

**Affiliations:** Buffalo, N. Y.


### Renal and Bladder Tuberculosis.[1]

By J. HENRY DOWD, M. D., Buffalo, N. Y.

THE subject of tuberculosis of the urinary organs has been studied so carefully during the past few years that it would seem that but little new could be added; nevertheless, considering that it is still in its infancy, the writer hopes to add a few points looking to the early recognition of the true condition and with the avoidance of irritation the patient's life may be prolonged and annoying symptoms greatly mitigated.

Involvement of the kidneys or bladder by a tubercular process occurs as follows: (1) Through the lymphatics; (2) through the blood channels; (3) by direct extension, continuity; (4) by direct infection; (5) congenitally.

In case of the kidneys it is fair to assume that infection occurs through any of the above avenues, not barring direct infection which may happen through catheterisation. On the other hand, with the bladder there is no doubt, in the great majority of cases, that involvement takes place by extension downward from the kidney; but it also may take place from direct infection and through the blood or lymphatics. Involvement of these organs assumes two phases,– the one rapidly progressive, the other very insidious, provided in the latter case that irritation or other experimental interference is avoided.

It is in this latter variety of cases that months or even years may pass before a positive diagnosis can be made. This assertion may be strengthened further by the fact that in a great majority of cases pulmonary complications are wanting; or, if the lungs are involved, it is of so mild a type that cough and other symptoms have been scarcely noticeable. In contradistinction to tuberculosis of other parts of the body but very little dependence can be placed in the appearance of the patients or their general condition, for it is here we find a well nourished individual with no apparent cachexia until late in the disease, by which time a general systemic infection is in progress. As regards the

---

1. Read at the semi-annual meeting of the Medical Society of the County of Chautauqua, at Jamestown, December 17, 1901.

organs under consideration it is safe to state that the kidneys are the starting point of the disease in the great majority of cases, and these are involved either primarily or secondarily from the lungs or other structures above.

On the other hand, although the foregoing is an indisputable fact, it is nevertheless equally a fact that in only the very smallest percentage of cases are appreciable symptoms present until the bladder is involved. Although in almost every case the bladder is involved when symptoms referable to it become evident, still occasionally it is found that although the first symptoms may refer to the viscus they are reflex and are only pointing out the trouble higher up. The age and a careful history is of the utmost consideration. In a very large percentage of cases the first symptoms make their appearance between 17 and 40. In a series of 28 cases reported as operated by Morris, 18 occurred between 20 and 30, and of the 3 cases operated between 30 and 35, the first symptoms appeared from 5 to 6 years previous, thus making 21 out of 28 occurring between 20 and 30.

Careful attention should be given the urine for, although repeated examinations may show no tubercle bacilli, pus, blood cells and albumin may be found where little or no trouble was expected.

Being aware of the close similarity of tuberculosis and other symptoms of simple inflammation of these organs, it will not be my purpose to repeat a group of well known symptoms, but to enumerate a few points that may be considered almost pathognomonic. These may be divided as follows: history, localised lumbar pain, bladder capacity, incompatibility between tuberculosis of the bladder and nitrate of silver, and the downward extension of the pathological process.

*History.*—Symptoms referable to the bladder and kidneys occurring between 15 and 35 years, especially in the male and eliminating venereal disease, stone or other instrumental interference, and the urine being passed in two glasses shows the same opacity in both, points to a tubercular involvement.

*Pain.*—Unilateral lumbar pains, more marked by deep pressure, accompanied frequently or occasionally by blood in the urine, in the absence of other well known causes thereof, is very suspicious of tuberculosis.

*Bladder Capacity.*—Diminished capacity, *i. e.*, below seven or eight ounces, with only appreciable fulness, is one of the most important symptoms of tubercular cystitis. It occurs early and in practically every case in contradistinction to the fact, that in old prostatics, for instance, where the viscus has suffered for years capacity remains about normal. This is accounted for by the inflammatory process involving not only the entire thickness of the mucous membrane, but also the connective tissue lying between the membrane and the muscles. As the case progresses even the muscles become involved. Capacity will be found to vary according to the time the bladder has been involved, I having seen contraction so marked that but one ounce of pure water could be used without causing the most intense suffering. The best method for ascertaining capacity is by the use of the Ultzmann syringe (capacity, 6 oz.) with the writer's attachment. With this the exact amount of fluid is measurable, the propelling thumb telling when the bladder will stand no more dilatation.

*Incompatibility between Silver Nitrate and Tubercular Inflammation of the Bladder.*—Although this drug is the most important, and I might say the only one with curative properties in simple inflammations, in tubercular conditions of the bladder it is not only contraindicated, but serves as a valuable aid in the differential diagnosis. I will grant that a silver solution of 1-20,000 will cause some uneasiness in a very mild, simple cystitis, or posterior urethritis, but where in this condition there may be frequency with some tenesmus, lasting from thirty minutes to two hours, in case of tubercular involvement the frequency, pain and spasm is very pronounced, and will last from eighteen to twenty-four hours. Blood may follow each urination.

*Downward Extension of the Pathological Process.*—Practically without exception tubercular involvement of the kidneys extend downward by continuity; furthermore the process is rapid. In every case where the kidney has been involved primarily, I have been able (in the male) to diagnosticate involvement of the ureter, prostate, vesicles or epididymi. In the female, the same is true as far as the ureters and bladder is concerned.

*Treatment.*—But little hope can be entertained for a cure in these conditions, for the very good reason that we rarely see the patient until the bladder or other parts are involved, and then there is that indisputable fact that in a goodly number, possibly every case, both kidneys are suffering. Providing there is no evidence of a tubercular process in the lungs or other organs, and we are reasonably sure the condition is unilateral, not having involved the bladder or other viscera to any extent, we are justified in attacking the kidney, removing the organ at once, or what is still safer and gives better results, nephrotomy, and as soon as the other kidney undergoes compensatory change the diseased one should be removed.

But little or no benefit results from nephrotomy or nephrectomy after the bladder is involved to any extent, excepting in case of the ureter becoming occluded, producing pyonephrosis, when operative procedures are called for. Operations on the bladder have been so unsuccessful that nothing can be advised until possibly late in the disease, when relief from pain and frequency may be obtained from a suprapubic cystotomy. The main dependence must be placed on environment and internal medication, with the avoidance of all local interference, which is merely experimental.

Although the same improvement will not be manifest as in disease of the lungs, there is no doubt that a change of climate with out-door life will be beneficial. In these conditions there is always more or less interference with perfect assimilation, therefore foods rich in oxalate of lime or nitrogen should be prohibited to a certain degree, yet not to an extent that systemic tone may be interfered with. Tonics are very beneficial at times, but the main dependence must be placed upon creosote or guaiacol.

Creosote is said to be quite irritating to the kidneys, yet if the valerianate (3 to 10 drop doses) is used this is overcome and the patient gets the benefit of the valerian. Local measures, such as washing the bladder, are contraindicated except in case of a mild infection being present and due to the use of unclean catheters. This condition can be easily and quickly removed by the use of silver in about 1-15,000 solution.

378 Franklin Street.

---

Pulmonary Embolism After Anesthetics.—According to Stengel, (*American Journal of the Medical Sciences*, August, 1901,) pulmonary embolism following the use of ether or chloroform as anesthetics is generally the result of preëxisting disease of the myocardium. It occurs more frequently after operations for uterine myomata than under any other circumstances, the sequence of events being: thrombus of the pelvic vessels, pulmonary embolus, pneumonia, death. Sometimes these events follow one another with astonishing rapidity. The rôle played by the heart in this process is that of a predisposing cause.

